# Validating a measure of motivational climate in health science courses

**DOI:** 10.1186/s12909-023-04311-3

**Published:** 2023-08-02

**Authors:** Brett D. Jones, Jesse L. M. Wilkins, Ásta B. Schram, Tehmina Gladman, Diane Kenwright, César A. Lucio-Ramírez

**Affiliations:** 1 School of Education, Virginia Tech, 1750 Kraft Drive (MC 0302), Blacksburg, VA 24061 USA; 2 School of Health Sciences, University of Iceland, Reykjavík, Iceland; 3 Dean’s Department, University of Otago, Wellington, Newtown, Wellington, New Zealand; 4 Department of Pathology and Molecular Medicine, University of Otago, Wellington, Newtown, Wellington, New Zealand; 5 School of Medicine and Health Sciences, Tecnológico de Monterrey, Monterrey, N.L., Mexico

**Keywords:** Motivational climate, Student motivation, Engagement, MUSIC model of academic motivation inventory, Affective assessment

## Abstract

**Purpose:**

The aim of the study was to examine the validity evidence for the 19-item form of the MUSIC Model of Academic Motivation Inventory (College Student version) within health science schools in three different countries. The MUSIC Inventory includes five scales that assess the motivational climate by measuring students’ perceptions related to five separate constructs: empowerment, usefulness, success, interest, and caring.

**Background:**

The 26-item form of the MUSIC Inventory has been validated for use with undergraduate students and with students in professional schools, including students at a veterinary medicine school, a pharmacy school, and a medical school. A 19-item form of the MUSIC Inventory has also been validated for use with undergraduate students, but it has not yet been validated for use with medical school students. The purpose of this study was to provide validity evidence for the use of the 19-item form in heath science schools in three different countries to determine if this version is acceptable for use in different cultures. If validated, this shorter form of the MUSIC Inventory would provide more differentiation between the Interest and Usefulness scales and could reduce respondent fatigue.

**Methodology:**

Cook et al’s [1] practical guidelines were followed to implement Kane’s [2] validity framework as a means to examine the evidence of validity through scoring inferences, generalization inferences, and extrapolation inferences. Students (n = 667) in health science schools within three countries were surveyed.

**Results:**

The results produced evidence to support all five hypotheses related to scoring, generalization, and extrapolation inferences.

**Conclusions:**

Scores from the 19-item form of the MUSIC Inventory are valid for use in health science courses within professional schools in different countries. Therefore, the MUSIC Inventory can be used in these schools to assess students’ perceptions of the motivational climate.

## Introduction

The motivational climate in a course has been defined as “the aspects of the psychological environment that affect students’ motivation and engagement within a course” [3]. Five aspects of the psychological environment have been shown to be especially important to students’ motivation and achievement: students’ perceptions of empowerment/autonomy, usefulness, success, interest, and caring (see the *Handbook of Motivation at School* [4] for how these variables relate to student motivation and achievement). These five student perceptions have also been associated with other important outcomes, such as effort [5–7], identification with a domain (e.g., engineering [8, 9]), and course and instructor ratings [3, 7, 10].

In many studies that have investigated all five aspects of the motivational climate, researchers have measured motivational climate using the MUSIC Model of Academic Motivation Inventory (referred to as the “MUSIC Inventory” [11]) because it includes one scale to measure each of the five aspects of the motivational climate. Many of the studies that have used the College Student version of the MUSIC Inventory have been conducted in undergraduate courses [12, 13]; however, some studies have included students in professional schools [14, 15]. Gladman et al. [16] were the first to examine the validity of the MUSIC Inventory in a medical school setting, and they determined that scores from the 26-item form of the MUSIC Inventory were valid for use with medical students. However, through factor analysis, they discovered that three of the Interest scale items cross-loaded with the Usefulness scale items. To better understand why this occurred, Jones and Wilkins [17] removed the three Interest scale items that cross-loaded and created a shorter (19-item) form of the inventory. They found that all of the items loaded on the intended scale factors and none of the items significantly cross-loaded on any of the other scale factors. Although the Jones and Wilkins [17] study provided validity evidence for the shorter (19-item) form, it was conducted with students in 32 undergraduate courses that included a variety of disciplines. Therefore, it is still unknown as to whether the 19-item form is valid for use with students in health science schools.

The purpose of the present study was to address these issues by examining the validity evidence for the shorter (19-item) MUSIC Inventory within health science schools in three different countries. Specifically, the research question for this study was: To what extent are the scores from the 19-item form of the MUSIC Inventory (College Student version) valid for use in health science courses within professional schools in different countries? If the shorter (19-item) form of the MUSIC Inventory is found to be valid in these settings, it would alleviate the problems of the cross-loading items documented by Gladman et al. [16], and it could reduce respondent fatigue, especially when the items are part of a survey that includes other questions.

## Background

The MUSIC Model of Academic Motivation Inventory [11] was developed to provide a multidimensional measure of the motivational climate in learning environments. The inventory measures five aspects of the motivational climate that align with the five components of the MUSIC Model of Motivation [18–20]: eMpowerment, Usefulness, Success, Interest, and Caring (MUSIC is an acronym for these dimensions). The original College Student version of the MUSIC Inventory is comprised of 26 items; however, a 19-item form was validated by removing seven of the items from the 26-item form [17]. Table 1 provides the following: the definitions of the constructs measured by each scale, other constructs that are related to each scale, and the number of items in each scale for the 26-item and 19-item forms. For each item, students rate their level of agreement on a 6-point Likert-format scale ranging from 1 to 6 (1 = *Strongly Disagree*, 2 = *Disagree*, 3 = *Somewhat Disagree*, 4 = *Somewhat Agree*, 5 = *Agree*, 6 = *Strongly Agree*). The items in each scale are averaged to produce a scale score that ranges from 1 to 6.

**Table 1:** Scales in the College Student Version of the MUSIC Inventory

MUSIC scale	Definitions	Related constructs	Items per scale	
	The degree to which a student perceives that:		26-item form	19-item form
Empowerment	they have control of their learning environment in the course	Autonomy [21]	5	4
Usefulness	the coursework is useful to their future	Utility value [22]	5	4
Success	they can succeed at the coursework	Expectancy for success [22]	4	4
Interest	the instructional methods and coursework are interesting (the definition in the 19-item form is: the instructional methods are interesting)	Situational interest [23]	6	3
Caring	the instructor cares about whether they succeed in the coursework and cares about their well-being	Caring [24]	6	4

The College Student version of the MUSIC Inventory has been validated for use with undergraduate students in many different types of courses, ranging from hard sciences to arts [3, 6, 7, 25–27]. It has also been validated for use with students in health science fields, including medical students [16], pharmacy students [15], and veterinary medicine students [14]. Jones and Wilkins [17] validated two shorter forms of the College Student version (including the 19-item form) with students in undergraduate courses.

Although the 19-item and 26-item forms of the MUSIC Inventory include the same items, there are two primary differences between these two forms. First, the 19-item form has seven items fewer than the 26-item form because it was created by removing seven items from the 26-item form [17]. Second, the Interest scale in the 26-item form assesses students’ interest in instructional methods and coursework, whereas the 19-item form assesses students’ interest in only the instructional methods. The reason that the 19-item form does not measure students’ interest in the coursework is because the three items that measured students’ interest in the coursework cross-loaded with the items in the Usefulness scale in the Gladman et al. [16] study. Gladman et al. [16] documented that removing these three items from the Interest scale had two effects: (1) it improved the fit indices and (2) the factor loadings of the individual items were higher on the intended factors. Similarly, Jones and Wilkins [17] found that removing these three items from the Interest scale produced fit statistics that were acceptable and similar to those reported in other studies of undergraduate students [7, 10]. Furthermore, the correlation between the Interest scale and the Usefulness scale for the 19-item form (*r* = .53) was less than the correlation between the Interest scale and the Usefulness scale for the 26-item form (*r* = .66) [17]. The better fit indices and the lower correlations between the Interest and Usefulness scales for the 19-item form were likely due to the fact that the three items assessing students’ interest in the coursework were measuring some aspect of their *individual* interest (i.e., their more enduring interest of the course topics over time) [23]. In contrast, their interest in the instructional methods may be situation dependent, and thus, serve as a measure of their *situational* interest (which can fluctuate over time from situation to situation) [28]. In sum, the Interest scale in the 19-item form of the inventory is a better measure of students’ *situational* interest because it is more distinct from their perceptions of usefulness than the 26-item form which also includes their perceptions of the coursework.

The purpose of the present study was to build on these prior studies by asking the question: To what extent are the scores from the 19-item form of the MUSIC Inventory (College Student version) valid for use in health science courses within professional schools? The answer to this question is not yet known because the 19-item form has not been investigated in health science courses. Although Gladman et al. [16] removed the three Interest scale items that assessed interest in the coursework, they did not remove items from the other scales to create the 19-item form of the inventory. Furthermore, Gladman et al. [16] used the College Student, *major/program level* version of the MUSIC Inventory to assess students’ perceptions of their medical school program and the courses that they had taken previously (and were enrolled in currently). Most studies have used the College Student version that asks students about their perceptions of one specific course [3, 6, 7, 25−27]. Therefore, the present study could be useful to validate the scores from the 19-item form in health science courses, and to do so with the version that asks questions in relation to one specific course (as opposed to their overall perceptions of many courses within a program). Finally, although the MUSIC Inventory has been translated to many different languages (see Jones [11] for all of the languages), the 19-item form has not been validated for use with any language other than English. In the present study, we sought to validate not only the English version, but also the Spanish and Icelandic translations of the inventory.

## Method

### Participants and procedures

Students in three health science schools from three different countries on three different continents participated in this study. Students were asked to complete an online survey in the middle of a course to provide feedback to instructors that could be used to improve the course. The survey results were provided to the instructors, and then the data were analyzed for the present study. In this section, we describe the students at each school separately because we conducted our analyses separately for each school.

In a medical school in New Zealand, students were asked to complete a survey in one fourth-year course (*n* = 95) and one fifth-year course (*n* = 102) that were taught by a different instructor. Over half of the fourth-year students at the university completed the survey (59 of 95 students; 62.1%) and more than a third of the fifth-year students completed the survey (39 of 102 students; 38.2%), for a total of 98 students (49.7% response rate) who participated in the study. Students reported their gender identity as: 51 (52.0%) females, 28 (28.6%) males, 2 (2.0%) non-binary, 3 (3.1%) preferred not to report, and 14 (14.3%) did not answer the question. Many of the students had lived most of their lives in New Zealand (*n* = 78, 80%), whereas 6 (6.1%) had spent more time in another country and 14 (14.3%) did not answer the question. The study was approved by the Human Ethics Committee at the participating university (Proposal #D22/063).

In a medical school in Mexico, students were asked to complete a survey in 16 different courses taught by 15 different instructors (one instructor taught two different courses). The number of students in each class ranged from two to 40 students. Of the 291 students in these 16 courses, 231 (79.4%) of the students completed the survey and were included in the study. The year of the students in medical school was as follows: 31 (13.6%) first year, 25 (11.0%) second year, 75 (32.9%) third year, 26 (11.4%) fourth year, 50 (21.9%) fifth year, 21 (9.2%) sixth year, and 3 (1.3%) who did not answer the question. Students’ self-reported gender identity was 163 (71.5%) females, 61 (26.8%) males, 4 (1.8%) another gender, and 3 (1.3%) did not report their gender. Most of the students had lived in Mexico most of their lives (*n* = 218, 95.6%), while 10 (4.4%) had lived most of their lives in another country and 3 (1.3%) did not answer the question. The study was not reviewed by an institutional review board (IRB) at the participating university because the data were initially collected as part of a workshop to improve instruction in the courses; it was not collected for the purposes of this study. The IRB at the institution of the first two authors determined that IRB review and approval was not required, in part, because the researchers were analyzing de-identified data after it had been collected for another purpose (IRB #22–798).

In a health science school in Iceland, 338 students in 16 courses (one of which was surveyed in two different years) completed a survey about their perceptions of their course. Six (37.5%) of the participating courses were undergraduate courses, 8 courses were graduate courses (50.0%), and 2 (12.5%) courses were available to both undergraduate and graduate students. The number of students in each class ranged from five to 132 students (there were 132 students total in the course that was taught twice). The participants self-reported their gender as 29 (8.6%) males, 308 (91.1%) females, and 1 (< 1.0%) did not provide a response. International regulations were followed with respect to informed participant consent for all aspects of the study. The National BioEthics Committee in Iceland does not receive applications for approval for this type of study because the survey did not contain any sensitive questions and students could choose to participate or not. However, as per Icelandic Regulations, the project was reported to the Icelandic National Data Protection Agency, and they listed the project in their publicized records on their webpage.

### Measures

The measures described in this section were included in an online survey written in English for the New Zealand university survey, in Spanish for the Mexican university survey, and in Icelandic for the Icelandic university survey.

#### The MUSIC Model of Academic Motivation Inventory (College Student version)

The College Student version of the MUSIC Model of Academic Motivation Inventory [11] was administered to students in university health science courses as described in the “Participants and Procedures” section. The survey included the 26-item form of the MUSIC Inventory for the New Zealand and Mexican universities, and the 19-item form of the MUSIC Inventory for the Icelandic university. During our analysis, we removed seven items from the 26-item version to create the 19-item form (the items we removed are noted by an asterisk in Table 2). The 26-item Spanish translation had been validated for use with undergraduate students previously [29]. The Icelandic translation had been validated for use with middle school students [30], but it had not been validated for use with university students. One of the Usefulness scale items (U2 = The coursework is beneficial to me) was inadvertently excluded from the Icelandic university survey; therefore, the Usefulness scale consisted of three items on the Icelandic survey.

**Table 2:** Items in the 26-item and 19-item Forms of the MUSIC Inventory

Scale	Items
Empowerment	* M1 = I have the opportunity to decide for myself how to meet the course goals. M2 = I have the freedom to complete the coursework my own way. M3 = I have options in how to achieve the goals of the course. M4 = I have control over how I learn the course content. M5 = I have flexibility in what I am allowed to do in this course.
Usefulness	U1 = In general, the coursework is useful to me. U2 = The coursework is beneficial to me. U3 = I find the coursework to be relevant to my future. * U4 = I will be able to use the knowledge I gain in this course. U5 = The knowledge I gain in this course is important for my future.
Success	S1 = I am confident that I can succeed in the coursework. S2 = I feel that I can be successful in meeting the academic challenges in this course. S3 = I am capable of getting a high grade in this course. S4 = Throughout the course, I have felt that I could be successful on the coursework.
Interest	* I1 = The coursework holds my attention. I2 = The instructional methods used in this course hold my attention. I3 = I enjoy the instructional methods used in this course. I4 = The instructional methods engage me in the course. * I5 = I enjoy completing the coursework. * I6 = The coursework is interesting to me.
Caring	* C1 = The instructor is available to answer my questions about the coursework. C2 = The instructor is willing to assist me if I need help in the course. C3 = The instructor cares about how well I do in this course. C4 = The instructor is respectful of me. C5 = The instructor is friendly. * C6 = I believe that the instructor cares about my feelings.

*Note*. Items with an asterisk were removed for the 19-item form.

#### Course effort

Students’ perceived course effort was assessed using the 4-item Course Effort scale [6], which measures the amount of effort that students believe that they are putting into a course. One of the items is, “I try my hardest to do very well in this course” (see Jones [11] for the complete scale). Students rate their effort on the same 6-point Likert-format scale that is used for the MUSIC Inventory. The reliability for the scale scores has been found to be good in other studies of undergraduate courses (α = 0.93, 0.87, 0.94, 0.83, and 0.79 in Jones [6]). In the present study, the Cronbach’s alpha value (0.89 for all three universities) and McDonald’s omega values (0.90 for New Zealand; 0.89 for Mexico and Iceland) were very good. One scale item (“In this course, I put forth my maximum effort”) was inadvertently excluded from the survey at the Icelandic university; therefore, the scale included only three items at that university.

#### Course and instructor ratings

In the New Zealand and Mexican universities, students rated the course and instructor on one item for each using the following Likert-format scale: 1 = *Terrible*, 2 = *Poor*, 3 = *Satisfactory*, 4 = *Good*, 5 = *Very Good*, 6 = *Excellent*. The course rating item was “My overall rating of the course,” and the instructor rating item was “My overall rating of the instructor for this course.” These two items have been used in other studies and have been shown to be associated with the MUSIC constructs [5, 10]. These course and instructor rating items were not included in the Icelandic university survey.

### Analysis

We used Kane’s [2] validity framework to validate the interpretations and uses of the 19-item MUSIC Inventory scores. To do so, we followed Cook et al’s [1] practical guidelines and examined three types of validity inference: scoring, generalization, and extrapolation. Table 3 summarizes the hypotheses and analyses related to each of these three types of validity inference. We conducted the analyses presented in Table 3 to provide validity evidence to support or refute each of these hypotheses.

**Table 3:** Hypotheses and Analyses Used to Provide Validity Evidence for Each Type of Validity Inference

Validity Inference	Definition	Hypotheses	Analyses
Scoring	The scores from the scales adequately capture students’ perceptions of the psychological construct	H1: The mean values and standard deviations on the scales of the 19-item inventory will be similar to the values on the scales of the 26-item inventory H2: Scales in the same inventory will demonstrate evidence of coherence and independence (i.e., the scales will not be highly correlated)	A1: Computing means and standard deviations for the scales in the 19- and 26-item inventories A2.1: Computing Pearson’s correlation coefficients to assess associations between scales in the 19-item inventory A2.2: Conducting factor analysis for all items in the 19- and 26-item inventories
Generalization	The items are representative of all theoretically possible items relevant to the construct	H3: The scale scores for the 19-item inventory will be highly correlated with scale scores for the 26-item inventory H4: Internal consistency analysis will indicate low error variance for each scale (i.e., Cronbach’s alpha and McDonald’s omega values will be acceptable)	A3: Computing Pearson’s correlation coefficients to assess associations between the 19- and 26-item inventory scales A4: Calculating Cronbach’s alpha and McDonald’s omega values for each scale
Extrapolation	The scores from the scales are related to other variables as anticipated	H5: Each scale score will be related to other variables as anticipated	A5: Computing Pearson’s correlation coefficients to assess associations between the scales and student effort, instructor ratings, and course ratings

*Notes*. References to the “scales” refer to the five scales of the MUSIC Inventory: Empowerment, Usefulness, Success, Interest, and Caring. Each hypothesis number aligns with the analysis with the same number.

## Results

### Evidence of validity through scoring inferences

We tested the first hypothesis for New Zealand and Mexico by comparing means and standard deviations for the 19-item form with those of the 26-item form. H1 was not tested in Iceland because the 26-item form was not administered there. The results presented in Table 4 show that the means and standard deviations were the same, or almost the same, across forms within the same university. All mean differences were less than or equal to 0.10. These findings provide evidence to support H1.

**Table 4:** A Comparison of Means and Standard Deviations for the 19- and 26-item Forms

Scale	19-item form		26-item form		Difference	
	*M*	*SD*	*M*	*SD*	*|ΔM|*	*|ΔSD|*
New Zealand						
Empowerment	4.46	0.83	4.47	0.82	0.01	0.01
Usefulness	4.82	0.99	4.82	0.97	0.00	0.02
Success	4.42	0.86	4.42	0.86	0.00	0.00
Interest	4.30	1.08	4.24	1.08	0.06	0.00
Caring	5.25	0.63	5.15	0.64	0.10	0.01
Mexico						
Empowerment	5.08	0.78	5.10	0.78	0.02	0.00
Usefulness	5.39	0.75	5.40	0.72	0.01	0.03
Success	5.35	0.71	5.35	0.71	0.00	0.00
Interest	5.13	0.98	5.16	0.92	0.03	0.06
Caring	5.68	0.53	5.63	0.56	0.05	0.03
Iceland						
Empowerment	4.06	0.97				
Usefulness	4.77	0.98				
Success	4.63	0.93				
Interest	4.04	1.26				
Caring	5.18	0.79				

*Note: N* = 98 for the New Zealand university; *N* = 231 for the Mexican university; and *N* = 338 for the Icelandic university.

The fact that the 19-item form produced mean scores similar to the 26-item form indicates that the 19-item form can be used in place of the 26-item form without a significant change in the interpretation of the motivational climate scores. Note that it is not productive to compare the mean scores across universities because students were enrolled in different courses at each university and the courses are not representative of all the courses at each university.

The second hypothesis was that the scales in the 19-item form would demonstrate evidence of coherence and independence; that is, the five MUSIC scales would not be highly correlated with one another. We set 0.71 as a “high” correlation between scales because it indicates that 50% (i.e., 0.71^2^) of the variance is shared between the scales. For the 19-item form, all 10 of the correlations between the scales within each university were less than 0.71 except for the one correlation between the Usefulness and Interest scales at the New Zealand university (*r* = .73) and the Mexican university (*r* = .74); the correlation between these two scales was 0.56 at the Icelandic university (see Table 5). The correlations ranged from 0.37 to 0.73 in New Zealand, 0.49 to 0.74 in Mexico, and 0.42 to 0.61 in Iceland. These Pearson’s correlation coefficients are similar to the values documented in studies of undergraduate students [6, 10, 17]. The correlation coefficients for the 19-item form are the same or similar to those in the 26-item form within the same university. (We discuss the correlations between forms in the next section with H3.)

**Table 5:** Pearson’s Correlations Among the MUSIC Model Components by Long and Short Forms

	M	U	S	I	C
New Zealand					
Empowerment	0.99	0.37	0.50	0.47	0.43
Usefulness	0.40	0.99	0.55	0.73	0.62
Success	0.53	0.56	1.00	0.60	0.43
Interest	0.50	0.77	0.62	0.97	0.53
Caring	0.49	0.62	0.47	0.59	0.96
Mexico					
Empowerment	0.98	0.61	0.53	0.63	0.55
Usefulness	0.63	0.99	0.58	0.74	0.56
Success	0.51	0.62	1.00	0.51	0.49
Interest	0.67	0.81	0.54	0.96	0.64
Caring	0.59	0.58	0.53	0.62	0.98
Iceland					
Empowerment		0.48	0.42	0.51	0.44
Usefulness			0.53	0.56	0.53
Success				0.61	0.58
Interest					0.60
Caring					

*Note*: Correlations for the 26-item form are below the diagonal; correlations for the 19-item form are above the diagonal. Correlations between the 26- and 19-item forms are on the diagonal.

The Pearson’s correlation coefficients provide evidence to support H2, with the exception that the coefficient between the Usefulness and Interest scales in New Zealand and Mexico were slightly higher than anticipated. Part of the reason for creating the 19-item form was to reduce the correlation between the Usefulness and Interest scales by removing the three items in the Interest scale that refer to “coursework.” Although the correlations were somewhat smaller between these scales in the 19-item form than the 26-item form (0.73 vs. 0.77 in New Zealand; 0.74 versus 0.81 in Mexico), the values for the 19-item form are still somewhat higher than anticipated.

To further investigate the relationships between the MUSIC scales for H2, we conducted a confirmatory factor analysis (CFA) for the correlated five-factor MUSIC model for the data from each university (see Table 6). Because of typical violations of multivariate normality for all three data sets, we used test statistics that are robust to non-normality (i.e., the MLM estimator in Mplus, Version 8.8; see Muthen and Muthen [31]). The model fit the data fairly well for both forms of the inventory [32, 33]. However, the Comparative Fit Index (CFI) was slightly lower than hypothesized (i.e., it was less than 0.95) [32] for the two Mexican forms and for the 26-item form in New Zealand. Nonetheless, the CFI value of 0.95 is not a strict cutoff value and must be interpreted within the context of the other fit indices [34]. The Standardized Root Mean Squared Residual (SRMR) values were all acceptable (i.e., they were less than 0.08) [32], and the values for the Root Mean Square Error of Approximation (RMSEA) were within an acceptable range (i.e., they were less than 0.07) [35]. Overall, the fit indices derived from the CFAs indicate that the factor structure associated with the 19-item form fit the data as well as, or better than, the factor structure associated with the 26-item form, especially the CFI values. These findings, combined with the findings from the Pearson’s correlation analyses provide evidence to support H2.

**Table 6:** Fit Indexes for the Confirmatory Factor Analyses

University	CFI	SRMR	RMSEA
New Zealand	0.970, 0.933	0.065, 0.064	0.051 (0.020, 0.073), 0.067 (0.052, 0.081)
Mexico	0.923, 0.909	0.073, 0.072	0.068 (0.057, 0.079), 0.065 (0.057, 0.073)
Iceland	0.970	0.045	0.048 (0.037, 0.058)

*Notes*: The first number in each cell corresponds to the 19-item form and the second numbers correspond to the 26-item form. Numbers in parentheses for RMSEA represent the 90% confidence interval. CFI = Comparative Fit Index; SRMR = Standardized Root Mean Squared Residual; and RMSEA = Root Mean Square Error of Approximation with 90% confidence interval. For the Icelandic analysis, missing data were imputed with individual scale means.

### Evidence of validity through generalization inferences

As a test of the third hypothesis, we compared the Pearson’s correlation coefficient between the 19-item and 26-item scales for each of the five MUSIC scales. The correlations were very high, as indicated by the values on the diagonals in Table 5 for New Zealand and Mexico. The values ranged from 0.96 to 0.99 for the Empowerment, Usefulness, Interest, and Caring scales; the correlation was 1.00 for the Success scale because the exact same items were used in both inventory forms. These results suggest that the rank of the scores in the 19-item and 26-item forms is similar. These findings provide evidence to support H3.

The fourth hypothesis was that the Cronbach’s alpha values and the McDonald’s omega values for each scale would be acceptable. We used the criteria that values greater than 0.7 were acceptable, between 0.8 and 0.9 were good, and greater than 0.9 were excellent [36]. The Cronbach’s alpha and McDonald’s omega values were exactly the same or only differed by 0.01 for each scale (see Table 7). All 50 values in Table 7 were greater than 0.80 (except for the alpha value for Empowerment in Mexico, which was 0.79), which indicated that the values were good or excellent. The differences between the 19- and 26-items forms within each country were minimal at 0.05 or less. The fact that the values for the 19-item scales are slightly lower than the 26-item scales is not unexpected because fewer items in a scale can reduce the reliability estimates [37].

**Table 7:** Cronbach’s Alpha Values and McDonald’s Omega Values for the Inventory Scales

Inventory form	Cronbach’s alpha values					McDonald’s omega values				
	M	U	S	I	C	M	U	S	I	C
New Zealand										
19-item	0.85	0.94	0.89	0.92	0.84	0.85	0.94	0.89	0.92	0.84
26-item	0.88	0.95	0.89	0.95	0.88	0.88	0.95	0.89	0.95	0.88
Mexico										
19-item	0.79	0.87	0.88	0.91	0.85	0.80	0.87	0.89	0.91	0.85
26-item	0.84	0.89	0.88	0.94	0.89	0.84	0.89	0.89	0.95	0.90
Iceland										
19-item^a^	0.84	0.88	0.90	0.91	0.88	0.85	0.89	0.90	0.91	0.88

*Note*. *n* = 98 for the New Zealand university scales; *n* = 231 for the Mexican university scales; for the Icelandic university, *n* = 332 for Empowerment and Interest, *n* = 333 for Success and Caring, and *n* = 337 for Usefulness.

^a^The 19-item form was administered, but one of the Usefulness scale items was missing; therefore, the alpha value for the Usefulness scale is based on three items instead of four.

The Cronbach’s alpha and McDonald’s omega values for each scale is remarkably similar across countries, indicating that the inventory produces reliable scores across these three cultures. These values are also similar to those reported in studies of undergraduate students, which typically range from 0.80 to 0.95 [6, 7, 10, 25, 26]. In summary, the results shown in Table 7 provide evidence to support H4.

### Evidence of validity through extrapolation inferences

The fifth hypothesis was that each scale score would be related to other variables as anticipated. To test this hypothesis, we correlated the MUSIC scales with students’ course effort, instructor rating, and course rating because these variables have been shown to be correlated in prior studies [3, 6]. (Note that students were not asked to provide instructor ratings and course ratings in Iceland.)

The correlations between the MUSIC scales and the three other variables (i.e., Effort, Instructor Rating, and Course Rating) were the same or very similar for the 19-item and 26-item forms within each university (see Table 8). For the 19-item version, the correlations between the MUSIC scales and Effort ranged from 0.24 to 0.70 in New Zealand, from 0.40 to 0.58 in Mexico, and from 0.27 to 0.56 in Iceland, representing medium (*r* = .30 to 0.49) or large (*r* = .50 or greater) effect sizes [38]. These effect sizes are similar to those documented in studies of undergraduate courses (*r* ranged from 0.17 to 0.59 [6, 7, 26]). The correlations between the five MUSIC scales and Instructor Ratings and Course Ratings represented mostly large effect sizes (Instructor Rating *r* ranged from 0.49 to 0.67 in New Zealand and from 0.36 to 0.72 in Mexico; Course Rating *r* ranged from 0.48 to 0.72 in New Zealand and 0.45 to 0.71 in Mexico). These effect sizes are very similar to those reported in studies of undergraduate students (*r* ranged from 0.41 to 0.69 for instructor ratings and 0.55 to 0.73 for course ratings [7]; *r* ranged from 0.44 to 0.70 for instructor ratings and 0.49 to 0.63 for course ratings [student-level variables] [10]). We conclude that these effect sizes provide evidence to support H5.

**Table 8:** Pearson’s Correlation Coefficients Between the MUSIC Scales and Effort, Instructor Rating, and Course Rating

	Effort		Instructor rating		Course rating	
Scale	19-item	26-item	19-item	26-item	19-item	26-item
New Zealand						
Empowerment	0.24	0.29	0.49	0.51	0.57	0.58
Usefulness	0.70	0.71	0.59	0.58	0.56	0.54
Success	0.41	0.41	0.54	0.54	0.54	0.54
Interest	0.58	0.66	0.67	0.66	0.72	0.72
Caring	0.49	0.48	0.62	0.63	0.48	0.49
Mexico						
Empowerment	0.40	0.43	0.53	0.54	0.57	0.59
Usefulness	0.53	0.54	0.53	0.54	0.66	0.67
Success	0.50	0.50	0.36	0.36	0.45	0.45
Interest	0.58	0.61	0.72	0.70	0.71	0.75
Caring	0.44	0.45	0.68	0.67	0.53	0.52
Iceland						
Empowerment	0.27					
Usefulness	0.41					
Success	0.56					
Interest	0.49					
Caring	0.39					

## Limitations and future research

One of the limitations of this study was that we did not compare the 19-item inventory scale scores with those of other established scales to provide further evidence of validity through extrapolation inferences. However, we did compare the 19-item inventory scale scores to the 26-item inventory scale scores, and the scales in the 26-item inventory have been successfully compared to other established scales [39]. Future research could compare the MUSIC Inventory with other scales, such as the Strength of Motivation for Medical School Questionnaire or the Academic Motivation Scale. One consideration in conducting this type of comparison is that “motivation” scales can measure different aspects of motivation that are not conceptually similar. For example, the Strength of Motivation for Medical School Questionnaire measures the motivation that new medical students have for training in medical school [40], which is a very different type of motivation than what is measured by the MUSIC Inventory. Similarly, the Academic Motivation Scale [41] assesses students’ motivation related to why they attend college, which is different from the course perceptions assessed by the MUSIC Inventory. Therefore, researchers interested in providing concurrent validity evidence would have to be careful in selecting the other “motivation” scales carefully to ensure that they assess psychological constructs similar to those assessed by the MUSIC Inventory.

Another limitation of this study was that we did not develop new items for the inventory. It may be possible to improve the inventory by adding new items or revising existing ones. For example, the Cronbach’s alpha value for the Usefulness scale in the 19-item form was quite high in New Zealand at 0.94, which may indicate redundancy between the items. It may be possible to eliminate some of these items or revise them slightly.

## Discussion and conclusion

The primary aim of this study was to answer the question: To what extent are the scores from the 19-item form of the MUSIC Inventory (College Student version) valid for use in health science courses within professional schools in different countries? Using Kane’s [2] validity framework, we followed Cook et al’s [1] practical guidelines and examined the evidence of validity through scoring inferences, generalization inferences, and extrapolation inferences. We proposed five hypotheses and our findings produced evidence to support all five hypotheses. Therefore, we conclude that the scores from the 19-item form of the MUSIC Inventory are valid for use in health science courses within professional schools in different countries.

The 19-item form of the MUSIC Inventory will likely be preferred by educators and researchers over the 26-item form because it is shorter, which can reduce respondent fatigue, especially when the inventory is included in a survey with other questions. Because the psychometric properties of the 19-item form are similar to those of the 26-item form, there are no disadvantages (from a technical perspective) to using the 19-item form instead of the 26-item form. One possible disadvantage of using the 19-item form is that the Interest scale measures students’ interest in only the instructional methods, not the coursework. The 26-item form of the MUSIC Inventory may be more advantageous to individuals who want to measure students’ interest in both the instructional methods and the coursework because the Interest scale includes three additional items related to the coursework.

The fact that the MUSIC Inventory is valid for use at the three different universities that participated in this study provides cross-cultural validity evidence for the inventory. The three universities are located in three different countries—New Zealand, Mexico, and Iceland— that are located in different parts of the world and have different cultural traditions. These findings also provide validity evidence for the MUSIC Model of Motivation [18–20] more broadly. That is, the evidence demonstrates that students within three different cultures can make distinctions between five aspects of the motivational climate (i.e., empowerment, usefulness, success, interest, and caring) within a course. Furthermore, as predicted by the MUSIC model, these five motivational climate constructs are related to students’ engagement (e.g., effort) and instructor and course ratings. Overall, this study contributes to the research related to the MUSIC model and inventory by providing additional contexts in which they can be used.

## Data Availability

The datasets used and analyzed during the current study are available from the corresponding author on reasonable request.
